# An experimental test of the Allee effect range limitation hypothesis

**DOI:** 10.1111/1365-2656.13389

**Published:** 2020-11-29

**Authors:** Samuel A. Merker, Richard B. Chandler

**Affiliations:** ^1^ Warnell School of Forestry and Natural Resources University of Georgia Athens GA USA

**Keywords:** Canada warbler, climate change, density dependence, population dynamics, range limits, social cues

## Abstract

1. Understanding how climate change impacts trailing‐edge populations requires information about how abiotic and biotic factors limit their distributions. Theory indicates that socially mediated Allee effects can limit species distributions by suppressing growth rates of peripheral populations when social information is scarce.
2. The goal of our research was to determine if socially mediated Allee effects limit the distribution of Canada warbler *Cardellina canadensis* at the trailing‐edge of the geographic range.
3. Using 4 years of observational data from 71 sites and experimental data at 10 sites, we tested two predictions of the socially mediated range limitation hypothesis: (a) local growth rates should be positively correlated with local density and (b) the addition of social cues immediately outside the trailing‐edge range boundary would result in colonization of formerly unoccupied habitat and increased growth rates. During the third breeding season, social cues were experimentally added at 10 formerly unoccupied sites within and beyond the species’ local range margin to determine if the addition of social information could increase density and effectively expand the species’ range.
4. No experimental sites were colonized after adding social cues and no evidence of Allee effects was found. Rather, temperature, precipitation and negative density dependence strongly influenced population growth rates.
5. Although theoretical models indicate that the presence of socially mediated Allee effects at species range boundaries could increase the rate of climate‐induced range shifts and local extinctions, empirical results from the first test of this hypothesis suggest that Allee effects play a minimal role in limiting species’ distributions.

## Graphical abstract

This is the first empirical test of the socially mediated Allee effects range limitation hypothesis. Although most range limitation hypotheses have been thoroughly investigated, the Allee effect range limitation hypothesis has not. Contrary to predictions from theoretical models, our results indicate that Allee effects play a negligible role in limiting species distributions.

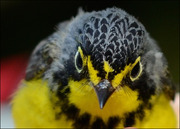

## 1 INTRODUCTION

The distributions of many species are shifting towards higher elevations and latitudes in response to climate change (Mason et al., 2015; Orihuela‐Torres et al., 2020; Parmesan, 2006; Parmesan & Yohe, 2003; Zuckerberg et al., 2009). Trailing‐edge populations near low‐elevation and low‐latitude range boundaries are predicted to experience strong negative impacts of climate change because these populations are often near their physiological limits and in contact with competitors and predators that are better adapted to encroaching climate conditions (Aitken et al., 2008; Cahill et al., 2014; Merker & Chandler, 2020b). Extinction of these populations could result in the loss of unique genetic diversity possibly leading to a decay of ecosystem function (Hampe & Petit, 2005). Understanding the mechanisms by which climate change will impact trailing‐edge populations requires information about the roles that abiotic and biotic factors play in limiting species distributions at low‐latitude, low‐elevation range boundaries (Cahill et al., 2014). One way in which biotic interactions can limit species distributions is through Allee effects, but the role of Allee effects in climate‐induced range shifts has received little attention outside theoretical contexts (Holt et al., 2004, 2005; Keitt et al., 2001; Schmidt et al., 2015).

Allee effects can result from biotic interactions that cause population growth rate to be positively correlated with population density (Allee et al., 1949; Kramer et al., 2009; Stephens et al., 1999). Examples include impaired mating opportunities and reduced pollination rates at low densities (Berec et al., 2018; Groom, 1998; Lande, 1987; Legendre et al., 1999). Prey species can also be impacted by Allee effects when confronted with subsidized predators capable of maintaining or increasing predation rates when the prey population is declining (de Roos et al., 1998; Keitt et al., 2001) Allee effects can increase extinction risk because population declines result in decreased growth rates, contrary to the stabilizing force that negative density dependence plays in regulated populations (Angulo et al., 2018; Bessa‐Gomes et al., 2004).

Theoretical work has demonstrated that Allee effects at the periphery of a species' range can result in stable range boundaries, even in the absence of other limiting factors, as long as Allee effects are not present in the interior of the range (Holt et al., 2004, 2005; Keitt et al., 2001). The models predict that low density populations near range margins can exhibit positive density dependence, which would prevent range expansion, whereas interior populations should be regulated via negative density dependence. These predictions have not been evaluated empirically.

**FIGURE 1:**
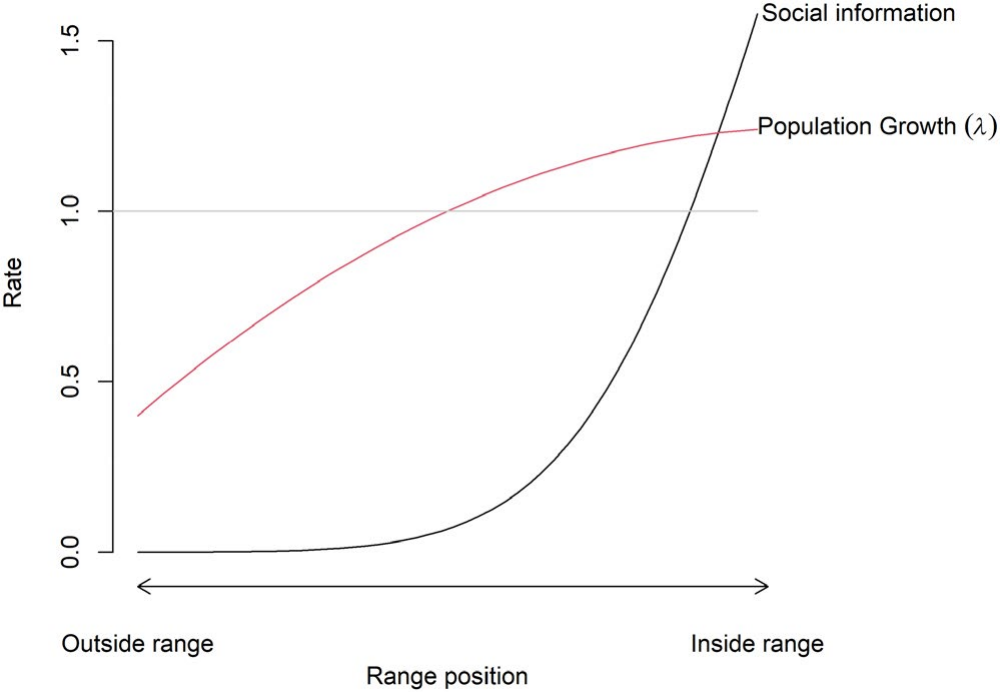
Conceptual figure demonstrating the socially mediated Allee effect range limitation hypothesis. As social information, like bird song, decreases at the edge of the species range so does population growth rate (*λ*). At the core of a species range social information is abundant and population growth rates are greater than one

Socially mediated Allee effects represent a mechanism by which positive density dependence near range margins could limit species’ distributions and contribute to climate‐induced range shifts (Angulo et al., 2018; Courchamp et al., 2008; Schmidt et al., 2015; Stamps, 1988). Socially mediated Allee effects occur when a population's growth rate depends on the availability of social cues. Many species utilize social cues when selecting breeding habitat because social cues can be a fast and often reliable way for individuals to determine if habitat is suitable (Ahlering et al., 2010; Betts et al., 2010; Schlossberg & Ward, 2004; Ward & Schlossberg, 2004). Several studies of passerines have shown that experimental introduction of conspecific song can cause individuals to colonize previously unoccupied habitat, regardless of habitat quality (Betts et al., 2008; Rushing et al., 2015; Ward & Schlossberg, 2004). For example, playing *Vireo atricapilla* (black‐capped vireo) song recordings during the post‐migration arrival and settlement periods in unoccupied habitat even in sites where reproductive performance ultimately proved to be poor (Ward & Schlossberg, 2004). These results suggest that the absence of social cues near range boundaries, where density is typically low and stochasticity is often high (Brown, 1984; Brown et al., 1996; Hampe & Petit, 2005), could prevent range expansion, and result in stable range boundaries as predicted by theoretical models (Figure 1).

Our objective was to determine if socially mediated Allee effects can limit species distributions at the trailing‐edge of a geographic range. Specifically, we assessed the hypothesis that trailing‐edge population range boundaries can be maintained by socially mediated Allee effects in which population growth rate decreases with local density as a result of decreasing amount of social cues available to inform habitat selection decisions (Figure 1). To evaluate this hypothesis, we tested the predictions that (a) local growth rate should be positively correlated with local density near the periphery of a range boundary and (b) that the addition of social cues immediately outside the trailing‐edge range boundary would result in colonization of formerly unoccupied habitat and increased population growth rates. We also evaluated the alternative hypothesis that range boundaries are shaped by the impacts of abiotic climate variables on population growth rates.

## 2 MATERIALS AND METHODS

### 2.1 Study system

We conducted our research on *Cardellina canadensis* (Canada warbler), a neotropical migrant passerine that breeds in the eastern United States and across Canada and winters in northern South America. As a result of long‐term population declines, it has been designated as threatened by the Committee on the Status of Endangered Wildlife in Canada (Hallworth et al., 2008; Hunt et al., 2017; Westwood et al., 2020). Canada warblers have a restricted breeding range in the southeastern United States, occurring only at high elevations in the Appalachian Mountains (Becker et al., 2012; Reitsma et al., 2020). The Canada warbler was selected as a focal species to test the socially mediated Allee effects hypothesis because its range boundary is clearly defined within the climate gradient that occurs in the southern Appalachian Mountains, and numerous studies have demonstrated that passerines use song as a breeding habitat selection cue (Betts et al., 2008; Rushing et al., 2015; Schlossberg & Ward, 2004; Ward & Schlossberg, 2004). Canada warblers are also known to respond to conspecific song during the breeding season. Additionally, because adult Canada warblers, individuals entering ≥ second breeding season, show strong site fidelity we anticipated that only young birds, individuals entering their first breeding season, would colonize our experimental sites (Reitsma et al., 2020).

We conducted field work near the trailing‐edge breeding boundary of *C. canadensis* in the United States Forest Service (USFS) Nantahala National Forest within and adjacent to the Coweeta Hydrologic Laboratory in southwestern North Carolina, USA. The area is characterized by steep topography ranging at an elevation of 660–1,590 m. Precipitation increases with elevation; ranging from 1,870 mm/year at low elevations to 2,500 mm/year at high elevations (Hwang et al., 2014). Daytime temperature tracks closely with elevation, becoming cooler at higher elevations. The study site is heavily forested with mixed hardwoods at low elevations transitioning to northern hardwood forests at higher elevations (Hwang et al., 2014; Webster et al., 2012). The understorey is primarily *Kalmia latifolia* (mountain laurel), *Rhododendron maximum* (big rhododendron) and *Vaccinium* spp. (Webster et al., 2012). Some areas have few shrubs and are relatively open from the forest floor to the canopy.

### 2.2 Data collection

**FIGURE 2:**
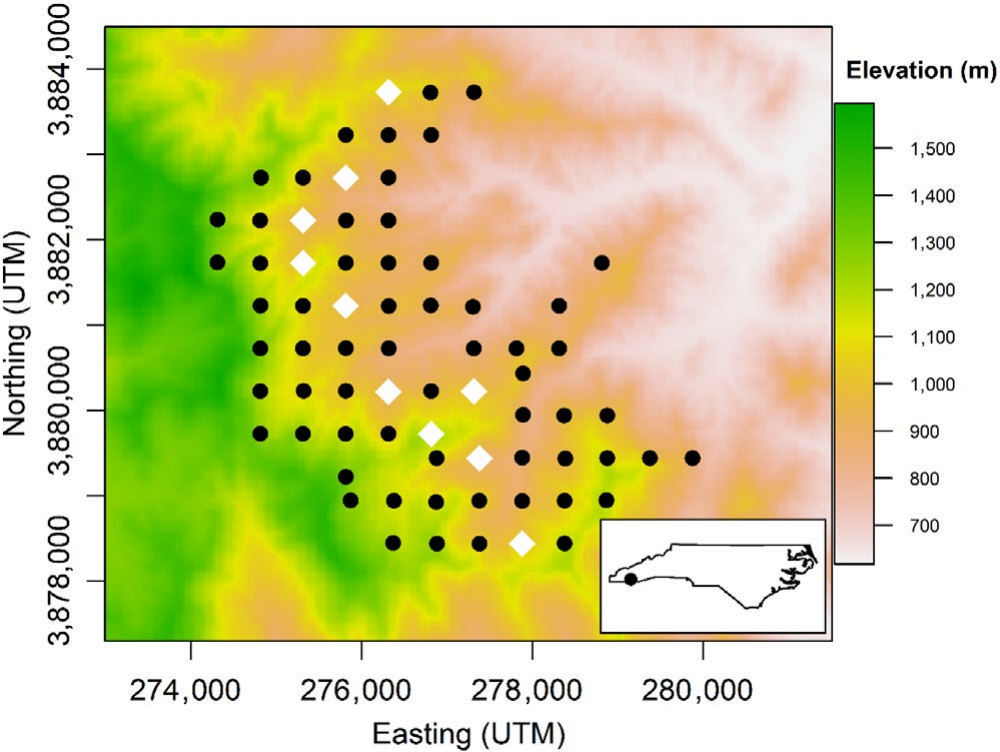
Point‐count survey locations in the Nantahala National Forest, North Carolina. Survey locations spanned the local range boundary of Canada warbler, which is restricted above 1,000 m elevation. Experimental sites are indicated by the white diamonds

We collected data on spatio‐temporal variation in Canada warbler abundance and growth rate using point‐count surveys at 71 locations from 2014 to 2017. Survey locations were positioned in a regular 500 m grid spanning the species local range boundary from approximately 800 to 1,400 m elevation (Figure 2). Canada warbler territory sizes are small, ranging from 0.2 to 1.5 ha making it unlikely that we double counted individuals between survey points (Reitsma et al., 2020). Each survey lasted 10 min, and consisted of four, 2.5‐min periods, during which every individual seen or heard within 100 m of the point was recorded. Surveys were conducted by expert observers able to identify Canada warblers by sight and vocalizations. Variables that could influence detection probability, including noise, time and wind were recorded during each survey on a 0–5 scale. Point‐count surveys were conducted on days with little or no precipitation, low wind and were limited to 06:00–11:00 hr, when birds are most active and likely to vocalize.

### 2.3 Conspecific attraction

We used methods similar to those of Ward and Schlossberg (2004) to experimentally add social information (i.e. broadcast song recordings) to previously unoccupied sites both within and outside the local range boundary of *C. canadensis* in southwestern North Carolina. The local range boundary and locations of unoccupied sites were identified using data from the first 2 years of point‐count surveys. Five experimental sites out of the 71 survey sites were selected within the range and five experimental sites were selected outside the range, below 1,000 m elevation (Figure 2; Chandler & Hepinstall‐Cymerman, 2016; Chandler et al., 2018). The remaining 61 sites served as controls that allowed us to account for extraneous sources of spatial and temporal variation in abundance and growth rate. All experimental sites included some combination of thick *R. maximum,* steep topography, and small first‐order streams all of which are key elements of Canada warbler habitat in this region (Reitsma et al., 2020).

We played recordings of *C. canadensis* song at 10 sites between 19 April and 7 May 2016 and at eight sites between 20 April and 11 May 2017 respectively. Recordings represented local Canada warbler dialect and were broadcast intermittently from 04:00 to 10:00 hr each day. Periods of silence and songs from other passerines from different families were included in playback to avoid habituation by Canada warblers and potential competitive interactions that may exist between this species and other warblers. Sample size decreased from 10 experimental sites to eight sites in 2017 due to destruction of playback units by *Ursus americanus* (American black bear). These dates are within the period when *C. canadensis* arrive on the breeding grounds in North Carolina, establish territories and begin nesting. Each playback unit consisted of a Raspberry Pi^®^ computer (Sony, Pencoed, Wales), a small amplifier, and a Yamaha^®^ outdoor speaker. Each unit was powered by two, 12 v sealed lead batteries. Playback was set to between 80 and 90 dB. Playback units could be heard from over 100 m (S. A. Merker, pers. obs.) and if neighbouring points also had playback it could not be heard between points. Playback units were designed, constructed and programmed by the University of Georgia's Instrument Fabrication and Design shop.

### 2.4 Climate data

**FIGURE 3:**
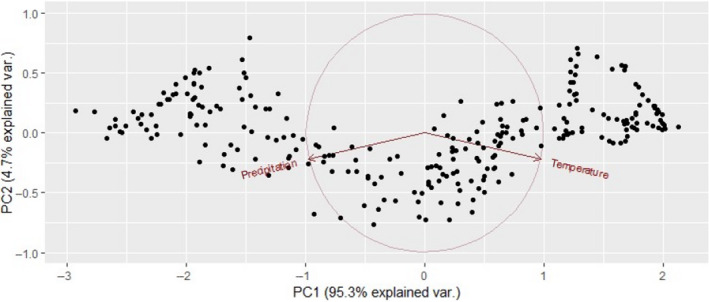
Principle components of PRISM temperature and precipitation in 30‐year normals. Principle component 1 was used to describe the climate gradient in the study area, and was included as a covariate in models of Canada warbler abundance. Negative values of PC1 represent cooler and wetter conditions. Positive values represent warmer and dryer conditions

We used publicly available precipitation and temperature data in the form of 30‐year normals from 1981 to 2010 (PRISM Climate Group, 2016). This data describe general climate patterns in the area. PRISM data were in raster format with a resolution of 800 m. Temperature and precipitation were highly correlated (*r* = −0.90), so we developed a single principle component to reflect the dominant climate gradient in the region. This principle component explained 95.3% of the variation (Figure 3).

### 2.5 Statistical analysis

We represented our hypotheses about the influence of Allee effects on spatial and temporal variation in abundance and growth rate using dynamic *N*‐mixture models that we fit to the point‐count data (Dail & Madsen, 2011; Royle, 2004). These models allow for inference on spatial and temporal variation in abundance while accounting for heterogeneity in detection probability, which can cause bias if ignored. Initial abundance (*N_i_*_,1_) at each site (*i* = 1, …, 71) was modelled as log‐linear function of climate:

$$\text{log(}\psi_{i})=\beta_{0}^{\left(\psi \right)}+\beta_{1}^{\left(\psi \right)}\;{\text{CLIMATE}}_{i},$$

$$N_{i,1}\;∼\;\text{Poisson}\;(\psi_{i})\text{.}$$

We modelled abundance in subsequent years (*t* = 2, 3, 4) as a function of the local growth rate (*λ_i_*_,_ *_t_*), which was influenced by climate, treatment (playback or no playback) and conspecific‐density in the preceding year:

$$\text{log(}\lambda_{i,t‐1})=\beta_{0}^{\left(\lambda \right)}+\beta_{1}^{\left(\lambda \right)}\;{\text{CLIMATE}}_{i}+\beta_{2}^{\left(\lambda \right)}\;{\text{TREATMENT}}_{i,t}+\beta_{3}^{\left(\lambda \right)}\;{\text{DENSITY}}_{i,t‐1},$$

$$N_{i,t}\;∼\;\text{Poisson(}N_{i,t‐1}\lambda_{i,t‐1})\text{.}$$

A negative value of *β*_3_ would be indicative of negative density dependence, whereas a positive value would suggest an Allee effect. Allee effects might not involve monotonic increases in the growth rate over a range of densities, and population densities may affect population growth rates differently under different climate conditions. Therefore, we considered eight additional growth rate models, some including quadratic effects of density and climate, and interactions between the density and climate (Table 1).

**TABLE 1:** Posterior summary statistics for parameters of the top model of Canada warbler abundance and growth rates in the southern Appalachian Mountains. Local growth rate was modelled as a function of climate variables, density dependence and the experimental addition of conspecific playback (treatment). Initial abundance was modelled as a function of climate variables. Detection was modelled as a function of wind, noise, Julian date and time of day

Process	Parameter	Mean	*SD*	Lower CI	Upper CI
Initial abundance	Intercept	−2.63	0.39	−3.44	−1.89
	Climate	−1.56	0.21	−1.99	−1.14
Growth rate	Intercept	−1.08	0.42	−1.96	−0.28
	Climate	−0.075	0.30	−1.37	−0.18
	Treatment	−1.46	0.99	−3.81	−0.04
	Density	−0.24	0.13	−0.54	−0.03
Detection	Intercept	0.37	0.15	0.05	0.68
	Wind	0.23	0.14	−0.05	0.53
	Noise	−0.57	0.19	−0.95	−0.17
	Date	0.51	0.24	0.04	0.99
	Time	−0.13	0.16	−0.45	0.17

We modelled detection probability in each year as a logit‐linear function of covariates that affect an observer's ability to detect an individual, including Julian date, time of day, noise and wind:

$$\text{logit(}p_{i,j,t})=\beta_{0}^{\left(p\right)}+\beta_{1}^{\left(p\right)}\;{\text{WIND}}_{i,j,t}+\beta_{2}^{\left(p\right)}\;{\text{NOISE}}_{i,j,t}+\beta_{3}^{\left(p\right)}\;{\text{DATE}}_{i,j,t}+\beta_{4}^{\left(p\right)}\;{\text{TIME}}_{i,j,t}.$$

We used diffuse normal distributions with a mean of 0 as priors for all regression coefficients (Appendix S1). Prior to model fitting all covariates were scaled and centred to ensure comparability. Models were fit using Markov chain Monte Carlo in a Bayesian framework. All analyses were conducted in R statistical software version 3.3.2 (R Core Team, 2019) and version 4.0.0 of Just Another Gibbs Sampler (JAGS; Plummer, 2017). Each JAGS model was run with three chains of 500,000 iterations each, a thinning rate of 20 and a burn‐in of 1,000. We used Watanabe–Akaike information criterion (WAIC) for model selection. We used Moran's *I* to test for spatial autocorrelation in our model residuals (Appendix S2). Additionally, we inspected model residuals for spatial autocorrelation by plotting them for each year (Appendix S3).

## 3 RESULTS

**FIGURE 4:**
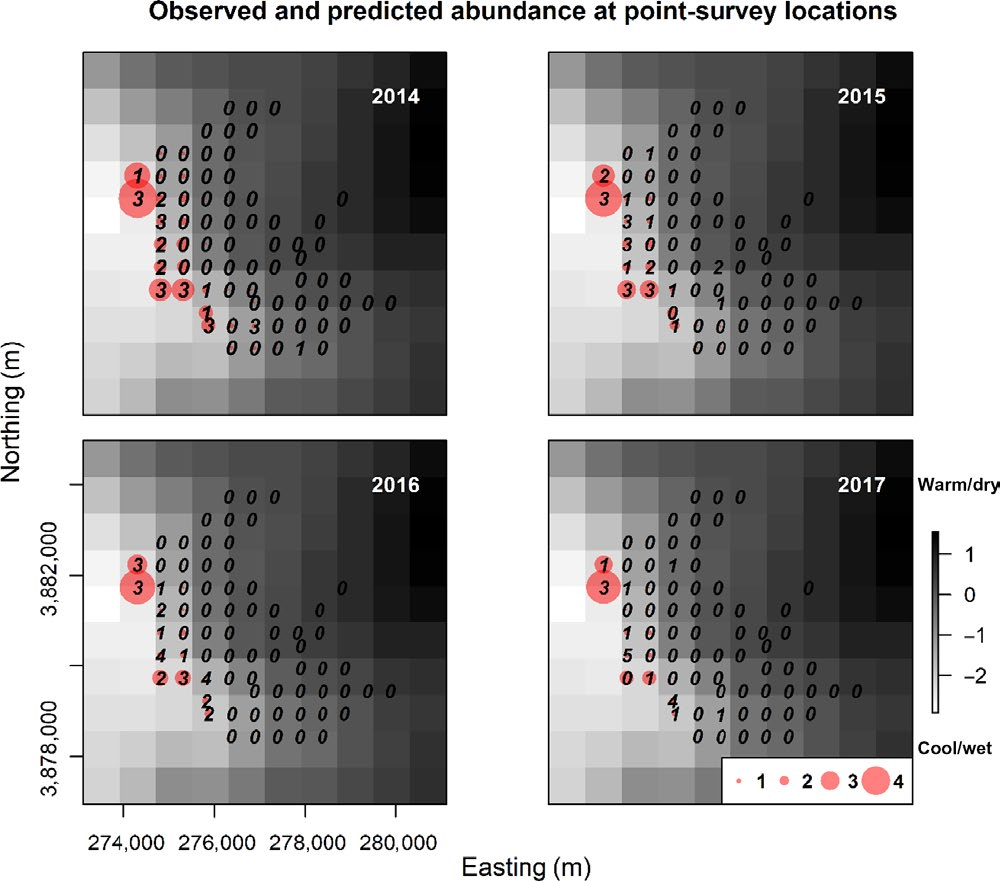
Maps of raw observed counts (integers) and model estimates (red circles) of Canada warbler abundance at survey locations in the Nantahala National Forest from 2014 to 2017. The background raster (800 m^2^) shows the climate gradient as a principle component of temperature and precipitation (PRISM Climate Group 2016). Lighter pixels represent cooler and wetter conditions at high elevations

We conducted 284 point‐count surveys between 2014 and 2017 (*n = *71 per breeding season). We detected 28, 28, 28 and 19 individuals in years 2014–2017 respectively. No more than five individuals were detected at a site and most sites initially occupied by *C. canadensis* in 2014 remained so in the following years (Figure 4). Sites at elevations lower than 1,000 m were rarely occupied by *C. canadensis*.

The model with the best WAIC score included a climate effect on initial abundance, and effects of climate, treatment and density on population growth rate (Table 1). The top model received 43.7% of the weight of all models considered. The second‐best model, similar to the top model, but including a weak quadratic effect of density, received 25.1% of the weight. All other models received <15% of the model weight. Only results from the top model are reported below.

**FIGURE 5:**
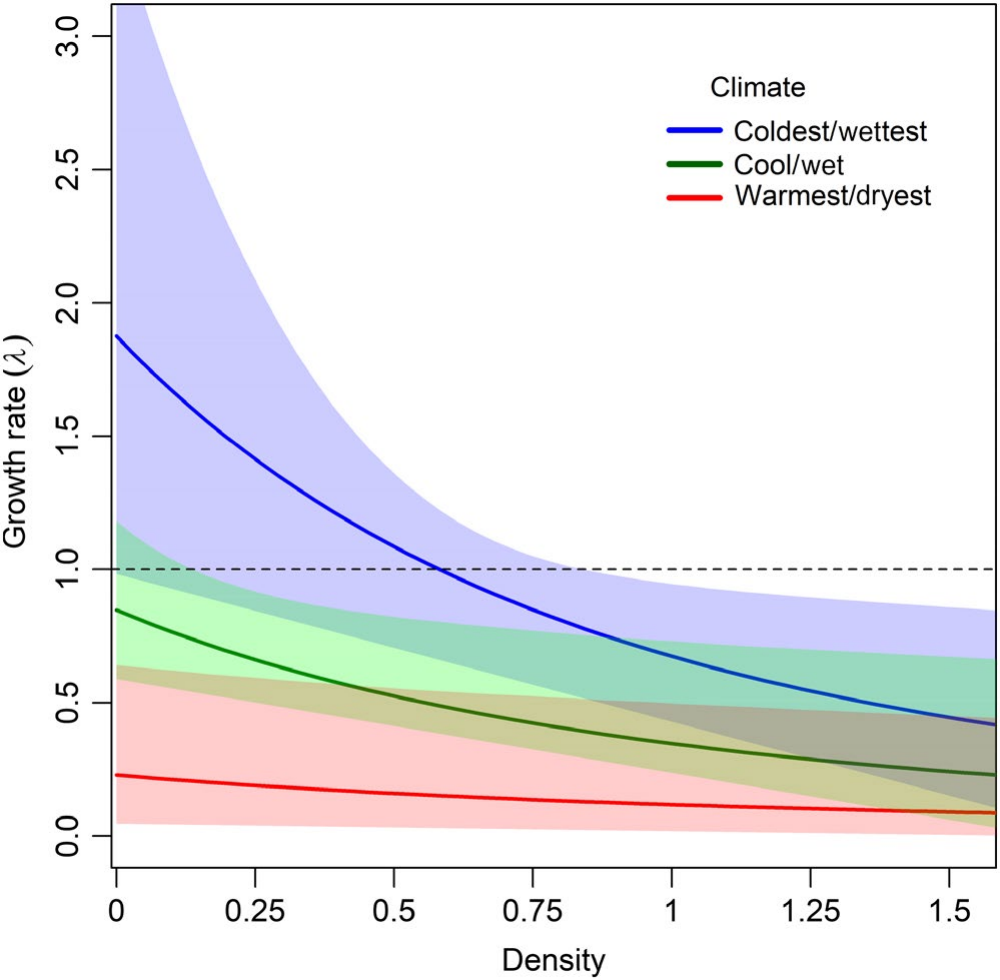
Model estimated density‐dependent population growth rates for Canada warblers *Cardellina canadensis* in three different climate conditions in the Nantahala National Forest, North Carolina. Climate conditions are derived from the principle component analysis and represent combinations of temperature and precipitation

Contrary to the socially mediated Allee effect range limitation hypothesis, population growth rate decreased as population density increased (Table 1; Figure 5). There was no evidence of a quadratic relationship between growth rate and density, indicating that density dependence was negative across the range of observed densities. Additionally, there was no evidence of a quadratic effect of climate on growth rates or density, and there was no evidence of an interaction between climate and density, suggesting that negative density dependence was maintained over the entire gradient of climate conditions.

In the warmer and drier conditions population growth rates were never greater than one (Table 1). Density and population growth rates of *C. canadensis* were highest at cooler and wetter sites, which generally occur at higher elevations (Figure 5). Population growth rates declined as average temperatures increased and average precipitation decreased. Growth rate was <1 across the range of observed densities in the warmer, drier conditions at lower elevations. In cooler wetter conditions, growth rate was >1 when density was low.

Detection probability during a single 2.5‐min period was 0.59 (0.51–0.72) at the average values of the covariates. The probability of detecting an individual over the 10‐min survey period was 0.972 (0.94–0.99). Detection of *C. canadensis* was negatively affected by ambient noise and positively affected by Julian date but it was not affected by wind or time of day (Table 2).

**TABLE 2:** Results of model selection using Watanabe–Akaike information criterion. The top model included climate effects on initial abundance (*N*_1_) and climate, treatment and density effects on population growth rate (*λ*). All models included wind, noise, date and time of day, as covariates of detection

Model	WAIC	ΔWAIC	Weight
*N*_1_ (climate) *λ* (climate + treatment + density)	1,708.00	0.00	0.44
*N*_1_ (climate) *λ* (climate + treatment + density^2^)	1,709.11	1.11	0.25
*N*_1_ (climate) *λ* (climate × density)	1,710.28	2.27	0.14
*N*_1_ (climate) *λ* (climate + density)	1,710.42	2.41	0.13
Global	1,712.75	4.74	0.04
*N*_1_ (climate) *λ* (.)	1,730.33	22.32	0.00
*N*_1_ (climate) *λ* (climate + treatment)	1,730.78	22.78	0.00
*N*_1_ (climate) *λ* (climate)	1,734.08	25.10	0.00
*N*_1_ (.) *λ* (.)	2,682.17	974.16	0.00

Consistent with the results from the observation data, experimentation indicated that Allee effects did not limit the distribution of *C. canadensis*. The experimental addition of playback did not result in the colonization of previously unoccupied habitat. No *C. canadensis* were detected at treatment sites where playback was added, regardless of elevation or climate suitability. Finally, we found no evidence of spatial autocorrelation in model residuals during any year of the study (Appendices S2 and S3).

## 4 DISCUSSION

Understanding the factors limiting species' distributions is one of the oldest pursuits in ecology, and it has become one of the most important subjects in efforts to conserve global biodiversity impacted by rapid environmental change (Cahill et al., 2014; Darwin, 1859; Gaston, 2009; MacArthur, 1972; Parmesan et al., 2005). Although most work has focused on abiotic limiting factors, theoretical and empirical work has demonstrated that biotic interactions can limit species’ distributions in the absence of abiotic constraints (Freeman et al., 2016; Freeman & Montgomery, 2016; Jankowski et al., 2010, 2013). However, the role of Allee effects in limiting species’ distributions is virtually unknown outside of theoretical contexts, and to our knowledge, this study represents the first empirical test of the socially mediated Allee effect range limitation hypothesis. Counter to predictions, we found no evidence that socially mediated Allee effects limit the distribution of *C. canadensis* at their warm‐edge range limit. Moreover, there was no evidence of positive density dependence near the range boundary or in the interior sites, indicating that no other process contributed to Allee effects.

Several hypotheses could explain the absence of Allee effects in our study system. The most likely explanation supported by our data is that the southern range limit of *C. canadensis* is shaped by climate conditions rather than Allee effects. Growth rates were closely correlated with local climate conditions, and these results support mounting evidence that climate and other abiotic factors can play a larger role than biotic interactions in limiting species distributions (Cahill et al., 2014; Hickling et al., 2006; Román‐Palacios & Wiens, 2020; Thomas, 2010). However, the mechanism by which temperature and precipitation affect this population is unclear. For example, it is unlikely that precipitation acts directly on individuals to limit fitness, but it may act indirectly by driving food availability for secondary and tertiary consumers like songbirds (Bolger et al., 2005; Holmes, 2011; Jones et al., 2003). Temperature is more likely to directly limit species distributions because some trailing‐edge populations may be at their thermophysiological limit (Root, 1988a, 1988b). As temperature increases, individual fitness may be reduced (Buckley & Huey, 2016; Lof et al., 2012), leading to a decrease in population growth rates. Increased understanding of how climate acts upon species distributions at the trailing edge of the range is needed to forecast climate change impacts, and future work should assess the impacts of annual variation in weather conditions on demographic parameters.

Because the southern Appalachian Mountain region is characterized by steep topography, the deep valleys and north facing slopes that make up much of the area may provide refuge by providing climatically suitable pockets to maintain these trailing‐edge populations. These climatically suitable pockets may not persist as climate change accelerates, especially if the frequency and duration of extreme weather events increase (IPCC, 2014). Furthermore, yearly variation in temperature and precipitation are often driven by major climate cycles such as El Niño and La Niña Southern Oscillations. These cycles can directly influence food resources for migratory birds and greatly affect local abundance and density in subsequent years (Rodenhouse et al., 2003, 2008; Sillett et al., 2000). Global environmental change may alter the timing and frequency of these climate cycles, with unknown consequences for trailing‐edge populations.

From a conservation standpoint, the absence of Allee effects is encouraging because Allee effects can increase extinction risk, making conservation intervention difficult relative to populations regulated by negative density dependence (Courchamp et al., 2008; Kramer et al., 2018). Allee effects near range boundaries could lead to stability as has been demonstrated theoretically, but they could also lead to rapid range contraction if environmental change and stochastic processes force small peripheral populations below the density threshold where positive density dependence occurs. Although we found no such phenomenon, additional research is needed to determine if this mechanism could explain range contractions of other species.

It is possible that the presence of conspecifics alone may not be a sufficient cue to elicit a habitat selection response. For example, an individual may identify possible breeding habitat through detection of conspecifics but may deem the habitat unsuitable once that habitat has been investigated further (Schmidt et al., 2015; Schmidt & Massol, 2019). This would run counter to previous studies that demonstrated that social cues can be used to attract individuals to low quality habitat. Another possible explanation for the absence of Allee effects is that *C. canadensis* may use social cues during a different season when selecting habitat. Our research was conducted during the arrival and settlement periods as with *Vireo atricapilla* (black‐capped vireo; Schlossberg & Ward, 2004; Ward & Schlossberg, 2004). However, recent studies have shown that some warbler species use social cues during the post‐breeding season, prior to migration, to select breeding habitat for the following season (Ahlering et al., 2010; Betts et al., 2008, 2010; Rushing et al., 2015). Future studies should attempt to assess the role that social information plays at multiple time periods throughout the breeding season, especially over different levels of habitat quality.

Although our results indicate that abiotic climate variables, not Allee effects, are the primary factor limiting trailing‐edge distributions, additional research is needed to determine the generality of our inferences. In systems where Allee effects do contribute to range limitation, the mechanism involved—socially mediated or otherwise—should be identified to guide conservation efforts.

## AUTHORS' CONTRIBUTIONS

S.A.M. and R.B.C. designed the study; S.A.M. collected the data and performed the analysis; S.A.M. wrote the first draft of the manuscript. Both authors contributed to revisions and gave final approval for publication.

## Supporting information

Supplementary Material

## Data Availability

Data available through Zenodo https://doi.org/10.5281/zenodo.4238700 (Merker & Chandler, 2020a).
