# Supplementary material for: An experimental test of the Allee effect range limitation hypothesis
Source: J Anim Ecol. 2020 Nov 29;90(3):585–93. doi: 10.1111/1365-2656.13389 (PMC7984094; doi:10.1111/1365-2656.13389)

Appendices for 'An experimental test of the Allee effect range limitation hypothesis'

Samuel Merker and Richard Chandler

Appendix 1: JAGS code

JAGS code for the dynamic N-mixture model including an interaction of climate and treatment effects on Canada warbler density and growth rate in the southern Appalachian Mountains of the United States.

```
model {

    ## Coefficients of model for initial abundance
    beta0 ~ dnorm(0, 1)
    beta1 ~ dnorm(0, 0.1)
    ## Coefficients of model for detection probability
    alpha0 ~ dnorm(0, 0.5)
    alpha1 ~ dnorm(0, 0.1)
    alpha2 ~ dnorm(0, 0.1)
    alpha3 ~ dnorm(0, 0.1)
    alpha4 ~ dnorm(0, 0.1)
    ## Coefficients of model for growth rate
    lambda0 ~ dnorm(0, 1)
    lambda1 ~ dnorm(0, 0.1)
    lambda2 ~ dnorm(0, 0.1)
    lambda3 ~ dnorm(0, 0.1)
    lambda4 ~ dnorm(0, 0.1)
    lambda5 ~ dnorm(0, 0.1)
    lambda6 ~ dnorm(0, 0.1)

    for(i in 1:nSites) {
      ## Model for year 1
      log(psi[i,1]) <- beta0 + beta1*climate[i]  ## psi is the expected value of N
      D[i,1] <- psi[i,1] / plotArea              ## D is the expected value of density
      DS[i,1] <- (D[i,1]-0.15)/0.25              ## Density (roughly) standardized
      N[i,1] ~ dpois(psi[i,1])                   ## Realized value of abundance
      ld.N.site.year[i,1] <- logdensity.pois(N[i,1], psi[i,1]) ## for WAIC
      for(j in 1:4) {
        ## Detection Probability
        logit(p[i,j,1]) <- alpha0 + alpha1*wind[i,j,1] + alpha2*noise[i,j,1] +
              alpha3*date[i,j,1] + alpha4*time[i,j,1]
        ## Count Data
        y[i,j,1] ~ dbin(p[i,j,1], N[i,1])
        ## Residuals
        resid[i,j,1] <- y[i,j,1]-N[i,1]*p[i,j,1]
        ## Log-densities to be used for WAIC
        ld.y.site.year.visit[i,j,1] <- logdensity.bin(y[i,j,1], p[i,j,1], N[i,1])
        ld.yN.site.year.visit[i,j,1] <- ld.y.site.year.visit[i,j,1]+ld.N.site.year[i,1]
```

```
      }
      ld.yN.site.year[i,1] <- ld.N.site.year[i,1]+sum(ld.y.site.year.visit[i,,1])
      resid.site.yr[i,1] <- mean(resid[i,1:4,1]) ## Average residuals at each site

      ## Model for years 2, 3, 4
      for(t in 2:T) {
        ## Growth Rate
        log(lambda[i,t-1]) <- lambda0 + lambda1*climate[i] + lambda2*treatment[i,1,t] +
            lambda3*DS[i,t-1] + lambda4*DS[i,t-1]^2 + lambda5*DS[i,t-1]*climate[i] +
            lambda6*DS[i,t-1]^2*climate[i]
        psi[i,t] <- psi[i,t-1]*lambda[i,t-1]
        D[i,t] <- psi[i,t] / plotArea
        DS[i,t] <- (D[i,t]-0.15)/0.25
        N[i,t] ~ dpois(psi[i,t])
        ld.N.site.year[i,t] <- logdensity.pois(N[i,t], psi[i,t])
        for(j in 1:nIntervals[t]) {
          logit(p[i,j,t]) <- alpha0 + alpha1*wind[i,j,t] + alpha2*noise[i,j,t] +
                alpha3*date[i,j,t] + alpha4*time[i,j,t]
          y[i,j,t] ~ dbin(p[i,j,t], N[i,t])
          resid[i,j,t] <- y[i,j,t]-N[i,t]*p[i,j,t]
          ld.y.site.year.visit[i,j,t] <- logdensity.bin(y[i,j,t], p[i,j,t], N[i,t])
          ld.yN.site.year.visit[i,j,t] <- ld.y.site.year.visit[i,j,t]+ld.N.site.year[i,t]
        }
        ld.yN.site.year[i,t] <- ld.N.site.year[i,t]+sum(ld.y.site.year.visit[i,,t])
        resid.site.yr[i,t] <- mean(resid[i,1:4,t])
      }
      ld.yN.site[i] <- sum(ld.yN.site.year[i,])
    }
    for(t in 1:T) {
      Ntotal[t] <- sum(N[,t])
    }
}
```

Appendix 2

An assessment of spatial autocorrelation in the residuals using Moran's I.

Moran's I

```
library(spdep)
cawa.resids <- read.csv("cawa-pb-resid.csv", row.names=1)
load("points.R")

## Coordinates (in the same site-order as resids)
coords <- as.matrix(all.points.xy)

dist.mat <- as.matrix(dist(coords))     # Distance matrix
weights <- ifelse(dist.mat>1000, 0, 1) # Neighbors defined as sites within 1 km
diag(weights) <- 0                      # Weight=1 for neighbors, 0 otherwise
weight.list <- mat2listw(weights)       # create neighbor list for spdep

Mi.1 <- moran.test(cawa.resids[,1], weight.list) #test of year 1 resids
Mi.2 <- moran.test(cawa.resids[,2], weight.list) #test of year 2 resids
Mi.3 <- moran.test(cawa.resids[,3], weight.list) #test of year 3 resids
Mi.4 <- moran.test(cawa.resids[,4], weight.list) #test of year 4 resids
```

Table 1. Moran's I for each year shows no indication of spatial autocorrelation in the model residuals.

Year	Moran's I	p-value
1	-1.343	0.910
2	0.250	0.401
3	-2.349	0.991
4	-1.762	0.961

Appendix 3

Model residuals at each survey point. The size of the point is proportional to the residual.

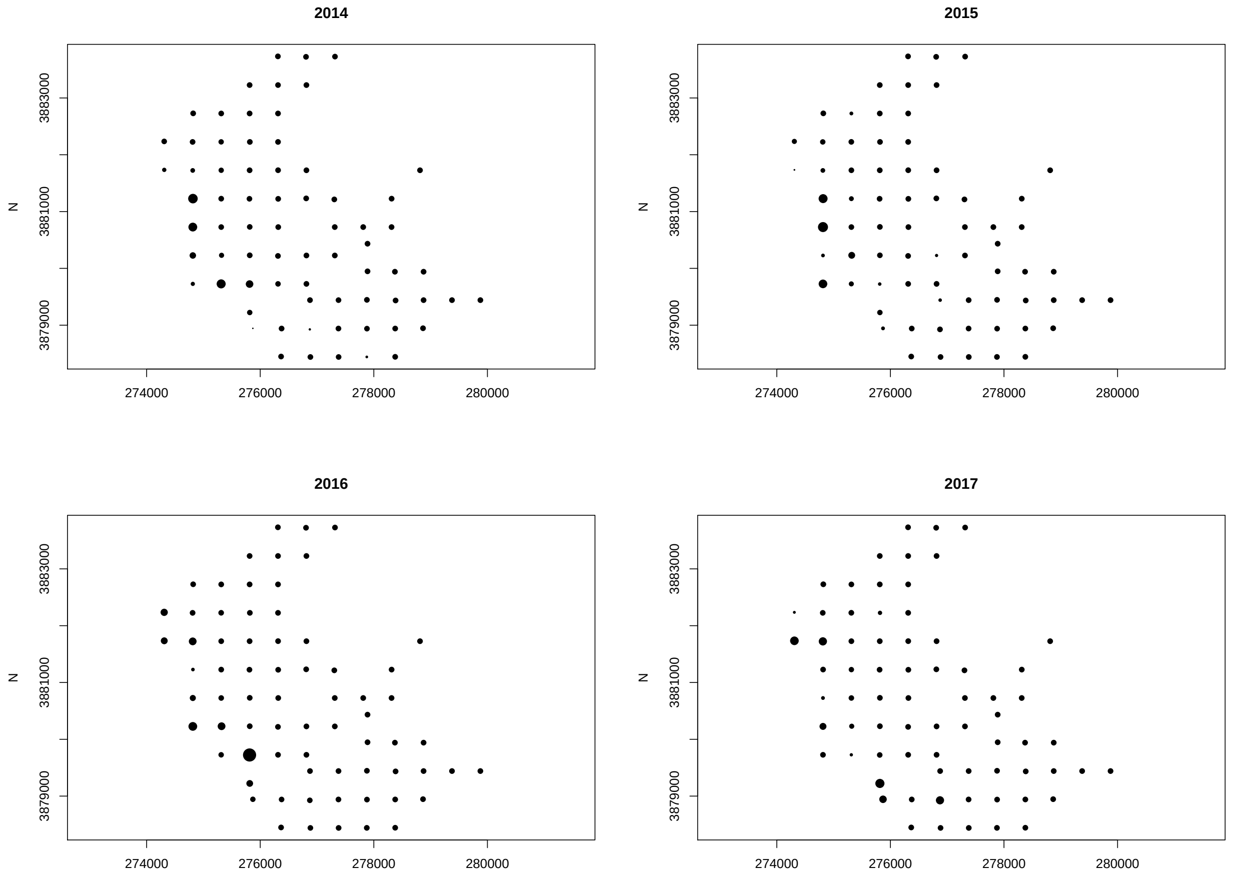

Appendix 4

Breeding range of Canada Warbler.

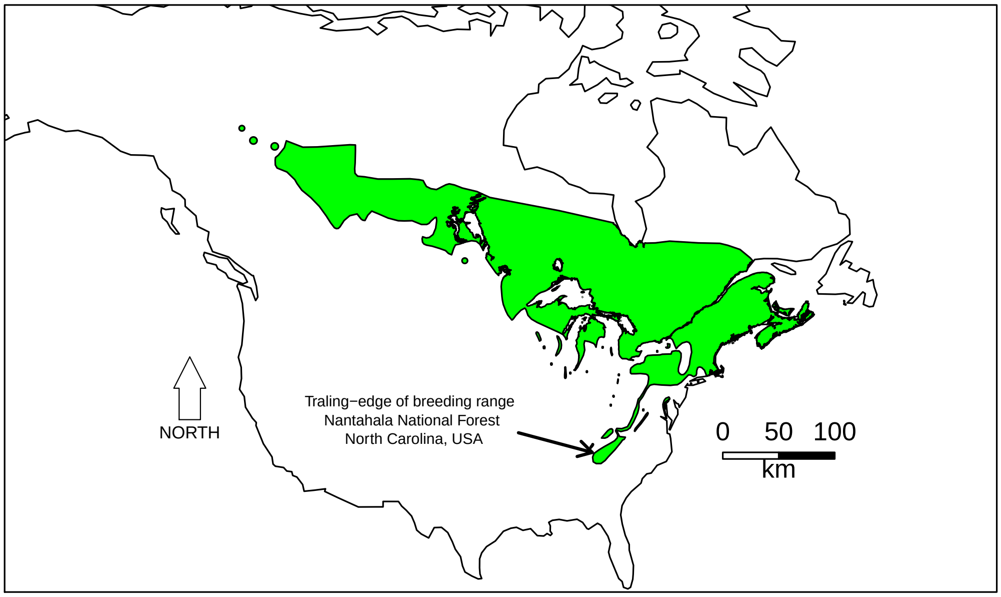

Supplement: Supplementary file 1 — Supplementary Material [file JANE-90-585-s001.pdf]
